# The application value of cerebrospinal fluid immunoglobulin in tuberculous meningitis

**DOI:** 10.1128/spectrum.00157-24

**Published:** 2024-04-26

**Authors:** Hua He, Jun Xu, Qin Peng, Yang Li, Ying Huang, Yan-Ling Zhang, Xiang Li

**Affiliations:** 1 Department of Infectious Disease, The Third People's Hospital of Kunming/Yunnan Clinical Medical Center for Infectious Diseases, Kunming, Yunnan, China; 2 Department of Radiology, The Third People's Hospital of Kunming/ Yunnan Clinical Medical Center for Infectious Diseases, Kunming, Yunnan, China; Children's National Hospital, George Washington University, Washington, DC, USA

**Keywords:** cerebrospinal fluid immunoglobulin, tuberculous meningitis, cryptococcal meningitis, diagnostic value

## Abstract

This article aims to study the value of cerebrospinal fluid (CSF) immunoglobulin in differential diagnosis, prediction, and prognosis of tuberculous meningitis (TBM). The clinical data of 65 patients with TBM in our hospital were collected, and 65 patients with cryptococcal meningitis (CM) were enrolled in 1:1 matching. Relevant data were collected for comparison. CSFs IgG [331.51 (164.85, 645.00) vs 129.00 (55.05, 251.00) ng/mL], IgM [22.38 (8.52, 40.18) vs 6.08 (2.19, 23.30) ng/mL], and IgA [64.11 (21.44, 115.48) vs 16.55 (4.76, 30.36) ng/mL] in the TBM group were higher than those in the CM group (*P* < 0.001). In the TBM group, after 24 weeks of treatment, the CSFs IgG, IgM, and IgA were significantly decreased, and the difference was statistically significant (*P* < 0.05). The predictive results of CSF immunoglobulin for TBM showed that IgG, IgM, and IgA all had some predictive value for TBM, and the combined predictive value of the three was the highest, with an area under the curve of 0.831 (95% CI: 0.774–0.881). Logistic regression analysis of CSF immunoglobulins and TBM prognosis showed that IgG [odds ratio (OR) = 4.796, 95% confidence interval (CI): 2.575–8.864], IgM (OR = 3.456, 95% CI: 2.757–5.754), and IgA (OR = 4.371, 95% CI: 2.731–5.856) were TBM risk factors for poor prognosis in patients. The levels of IgG, IgM, and IgA in CSF were positively correlated with the severity of cranial magnetic resonance imaging (MRI) in TBM patients (*R*^2^ = 0.542, *F* = 65.392, *P* < 0.05). CSFs IgG, IgM, and IgA can be used as a routine monitoring index for TBM patients, which has a certain reference value in differential diagnosis and efficacy evaluation.

**IMPORTANCE:**

In clinical practice, physicians can determine the physical conditions of patients based on the levels of cerebrospinal fluids (CSFs) IgG, IgM, and IgA. Higher levels of CSFs IgG, IgM, and IgA suggest more possibility of tuberculous meningitis and worse prognosis and magnetic resonance imaging manifestations.

## INTRODUCTION

Tuberculous meningitis (TBM) is a non-suppurative inflammatory disease of the membranes around the brain and spinal cord caused by *Mycobacterium tuberculosis* (Mtb) infection in the subarachnoid space, with the highest morbidity and mortality among all tuberculosis (TB) (1). Individuals at any age, especially children and untreated adults co-infected with human immunodeficiency virus (HIV), can be infected with Mtb, and the incidence of serious clinical outcomes, including death, is about 50% (2). Studies have shown that a large number of patients with TBM are not definitely diagnosed, and the likely causes of death from this disease include failure to provide rational treatment (3). The improvement in clinical outcomes depends on early diagnosis and early use of appropriate therapeutic doses of drugs for treatment (4).

The diagnosis of TBM is complicated. The current diagnosis of TBM is primarily based on the medical history, clinical manifestations, dynamic cytology of CSF, and brain and lung imaging examination. The detection rate of Mtb in blood, serous cavity effusion, or CSF, however, is very low. Moreover, there are difficulties in the identification of clinical symptoms (5), making the definite diagnosis of TBM more challenging.

The clinical manifestations of progressive TBM have been comprehensively summarized (6), and the diagnosis of this disease is often more evident when the symptoms of disease progression are present. The rapid progression of TBM will affect the outcomes of this disease. Due to the various genotypes of microorganisms infected (7), drug resistance (8), HIV co-infection (9), and Bacille Calmette-Guérin (BCG) vaccination status (10), the manifestations of TBM are inconsistent. TBM, which is curable at an early stage, can progress to coma, opisthotonos, and even death if it is not definitely diagnosed. Therefore, it is vitally important to identify TBM based on the early atypical symptoms.

Bacterial culture is considered the gold standard for the diagnosis of central nervous system infection (CNSI), but it is time-consuming and complicated to perform and has a low positive rate (11). The application of PCR technology can help clinical diagnosis quickly, but it is easy to appear false positive (12). The application of molecular biological methods for antibody detection also fails to provide a stronger basis for the clinic due to their low positive rate (13). Novel molecular assays and next-generation sequencing technologies have effectively enhanced detection capabilities, but there are sensitivity and specificity problems (14). Therefore, routine biochemical and cytological examinations of cerebrospinal fluid (CSF) are still the main method and important basis for the diagnosis of CNS infectious diseases.

The main source of CSF immunoglobulins (CSF Igs) includes those locally synthesized in activated immune cells after the CNS is infected and those entering the CSF from the blood when the blood-brain barrier (BBB) is altered (15). When the CNS is infected, the local synthesis of Igs increases, and the permeability of the BBB also changes, resulting in an abnormal increase in CSF Igs. The degree of CNS damage can be indirectly evaluated by the level of CSF Igs. Three types of Igs, including IgG, IgA, and IgM, can be generally detected in CSF. Studies have shown that changes in CSF Igs are more significant in patients with TBM compared with those in patients with CNS infections caused by other pathogens (16, 17). Detection of CSF Igs is helpful for the diagnosis of early TBM. On this basis, the present study was designed to investigate the value of CSF Igs in the differential diagnosis of TBM.

## STUDY SUBJECTS AND METHODS

### Study subjects

Sixty-five patients who were diagnosed with TBM at our hospital between January 2020 and December 2021 were retrospectively collected by convenience sampling and included in the TBM group. Sixty-five patients with cryptococcal meningitis (CM) who were admitted to the same hospital during the same period were selected in a ratio of 1:1 as the control group by propensity score matching, with matching factors of sex, age, body mass index (BMI), and underlying diseases. Inclusion criteria included patients (i) with an age of ≥18 years old, (ii) with effective anti-TB treatment for those included in the TBM group, and (iii) without other infectious diseases of the nervous system. Exclusion criteria included patients (i) co-infected with HIV, (ii) complicated with organic lesions of other important organs, (iii) who were not newly diagnosed, and (iv) complicated with major blood diseases. The study was approved by the ethics committee of our hospital.

### Methods

TBM was diagnosed according to the criteria described by Boyles et al. (18) and Huynh et al. (19), i.e., Mtb was isolated from CSF, or (i) clinical manifestations of fever, headache, vomiting, meningeal irritation sign, or focal neurological impairment; (ii) an increased white blood cell (WBC) count, increased protein content by quantitative measurement, and a decreased glucose and chlorides in CSF; and (iii) negative India ink staining and negative gram bacterial and fungal culture in CSF. A definite diagnosis was made if the above three criteria and one or more of the following three criteria were met: (i) basal cistern exudation, hydrocephalus, or cerebral infarction on cranial computed tomography or magnetic resonance imaging (MRI), (ii) presence of extracranial TB such as pulmonary TB, and (iii) effective anti-TB treatment.

CM was diagnosed according to the 2018 Chinese expert consensus on the diagnosis and treatment of CM (20), i.e., positive cryptococcal culture, positive India ink staining, and/or positive cryptococcal antigen test in CSF in patients with meningitis.

Clinical symptoms and signs of the patients were dynamically evaluated during the treatment, and the results 24 weeks after treatment were used as the final outcomes. Efficacies were classified as (i) cured: the clinical symptoms (fever, consciousness disorder, and cranial nerve damage) and signs (meningeal irritation sign and pathological sign) were completely resolved, CSF tests were normal (normal routine CSF biochemistry, negative acid-fast bacilli in CSF smears, and negative MTB culture of CSF), and the lesions were basically absorbed or calcified on cranial MRI; (ii) markedly effective: the clinical symptoms, part of meningeal irritation, and pathological signs, and the results of CSF tests were improved, and the lesions were partially absorbed on cranial MRI; (iii) ineffective: the clinical symptoms and meningeal irritation and pathological signs and the results of CSF tests did not change and even worsened from those before treatment, and the lesions were not absorbed and progressively increased and/or severe basicranial adhesion seen on cranial MRI; and (iv) death: the condition progressively aggravated, worsened, and led to death during the treatment. Due to the long course of TBM treatment (at least 12 months) (21), 24 weeks was not enough for the treatment of this disease. Therefore, efficacies that are evaluated as cured and markedly effective were classified as effective, with a favorable prognosis, and those evaluated as ineffective and death were classified as ineffective, with a poor prognosis.

CSF was collected from all patients via lumbar puncture and placed in three tubes, with 2 mL per tube. In addition, fasting venous blood was extracted and placed in three tubes, with 3 mL per tube. Blood samples were centrifuged at 3,500 rpm for 15 min, and then serum was collected. Using matching reagents, measure the concentrations of IgG, IgA, and IgM in CSF and serum using immunoturbidimetry on the Beckman Kurt IMMAGE 800 protein analyzer.

CSF MTB culture: using BACTECTMMGITTM960 fully automatic mycobacterium detection system, culture for six cycles; CSF Xpert MTB/RIF: the MTB rpoB gene and mutation detection kit (MTB/RIF Assay) was used, and real-time fluorescence PCR was used. MRI using co-imaging uMR780 3.0T.

### Data collection

General data and levels of CSFs IgG, IgM, and IgA before treatment were collected from the two groups of patients. In the TBM group, the level of CSF Igs in 24 weeks of treatment, the results of pathogenic examination of TB in CSF, CSF MTB culture, CSF XpertMTB/RIF detection, and the cranial MRI manifestations before treatment were collected.

### Statistic analysis

SPSS 26.0 statistical software was used for statistical analysis, and the Kolmogorov–Smirnov method was used for the normality test. Measurement data with normal distribution were presented as *x* ± *s*, and the mean values were compared between groups using the *t* test; those with skewed distribution were presented as *M* (*Q*1, *Q*3), group data were compared using the Mann–Whitney *U* test, and paired data were compared using the Wilcoxon signed rank test. Enumeration data were presented as frequencies (*n*) or rates (%), and *χ*^2^ test was used for comparison. The receiver operating curve was used to investigate the predictive value of CSF Igs for the development of TBM. The correlation between CSF Igs and prognosis of TBM was analyzed using a logistic regression analysis, and the correlation between CSF Igs and cranial MRI manifestations in TBM patients was analyzed using multivariate linear regression. All tests were two-sided, with a significance level of *α* = 0.05.

## RESULTS

### General data

There were 65 patients in the TBM group, including 37 males and 28 females, with a mean age of 30.29 ± 11.86 years old, a BMI of 23.47 ± 2.38 kg/m^2^, and a mean disease course of 20.45 ± 17.61 days, and 65 patients in the control group, including 40 males and 25 females, with a mean age of 30.29 ± 11.88 years old, a BMI of 24.38 ± 2.67 kg/m^2^, and a mean disease course of 18.92 ± 16.41 days. Both groups of patients who were included in the study did not receive BCG vaccination. Statistically significant differences between the two groups of patients were observed in CSF IgG [331.51 (164.85, 645.00) vs 129.00 (55.05, 251.00) ng/mL, *Z* = −4.845, *P* < 0.001], CSF IgM [22.38 (8.52, 40.18) vs 6.08 (2.19, 23.30) ng/mL, *Z* = −3.852, *P* < 0.001], and CSF IgA [64.11 (21.44, 115.48) vs 16.55 (4.76, 30.36) ng/mL, *Z* = −5.419, *P* < 0.001]. There were no statistically significant differences between the two groups of patients in sex, age, BMI, disease course, extracerebral lesions, chronic diseases, consciousness disorder, cranial nerve palsy, peripheral nerve dysfunction, CSF albumin level, serum albumin level, CSF WBC count, CSF glucose, CSF chloride, CSF neutrophil%, and CSF lymphocyte% (Table 1).

**TABLE 1:** Comparison of general data between the two groups^*a*^

Items	The TBM group (*n* = 65)	The control group (*n* = 65)	*t*/*χ^2^*/*Z*	*P*
Sex (M/F)	37/28	40/25	0.287	0.592
Age (years, *x* ± *s*)	30.29 ± 11.86	30.29 ± 11.88	0.591	0.558
BMI (kg/m^2^, *x* ± *s*)	23.47 ± 2.38	24.38 ± 2.67	−0.673	0.505
Duration of TBM (days, *x* ± *s*)	20.45 ± 17.61	18.92 ± 16.41	−0.258	0.798
Extracerebral lesions (*n*)	2	3	0.208	0.648
Chronic diseases (*n*)				
Hypertension	7	11	1.032	0.310
Diabetes	9	8	0.068	0.795
Consciousness disorder (*n*)	15	10	0.746	0.388
Cranial nerve palsy (*n*)	10	8	0.258	0.612
Peripheral nerve dysfunction (*n*)	12	7	1.541	0.214
CSF albumin (g/L, *x* ± *s*)	1.21 ± 0.54	1.24 ± 0.31	1.483	0.152
Serum albumin (g/L, *x* ± *s*)	36.46 ± 7.69	35.47 ± 6.79	0.062	0.804
CSF IgG (ng/mL, *x* ± *s*)	331.51 (164.85, 645.00)	129.00 (55.05, 251.00)	−4.845	<0.001
CSF IgM (ng/mL, *x* ± *s*)	22.38 (8.52, 40.18)	6.08 (2.19, 23.30)	−3.852	<0.001
CSF IgA (ng/mL, *x* ± *s*)	64.11 (21.44, 115.48)	16.55 (4.76, 30.36)	−5.419	<0.001
CSF WBC (×10^6^/L, *x* ± *s*)	183.49 ± 120.78	191.38 ± 125.39	0.178	0.859
CSF glucose (mmol/L, *x* ± *s*)	2.43 ± 1.21	2.21 ± 1.13	−1.418	0.164
CSF chloride (mmol/L, *x* ± *s*)	107.23 ± 8.65	103.47 ± 5.67	1.981	0.159
CSF neutrophils% (*x* ± *s*)	33.54 ± 14.92	35.47 ± 16.39	0.045	0.883
CSF lymphocytes% (*x* ± *s*)	68.39 ± 17.39	69.48 ± 18.38	1.159	0.257

^*a*^ BMI, body mass index; TBM, tuberculous meningitis; CSF, cerebrospinal fluid; WBC, white blood cell.

Among the 65 cases of TBM, 28 were confirmed cases: among them, 27 cases of MTB were detected in CSF culture, 4 cases of bacteria were detected in CSF acid-fast bacterial smear (4 cases showed MTB in later CSF culture without evidence of non-TB mycobacterium infection), and 8 cases were positive for Xpert MTB/RIF in CSF (7 cases showed MTB in later CSF culture); 37 cases were clinically diagnosed, including 22 cases with an average tuberculin skin test (TST) diameter ≥15 mm and 35 cases with positive interferon-γ release assay (IGRA) (Table 2).

**TABLE 2:** Etiology and immunology detection of TB in TBM patients

General information	*n*	%
TST		
≥5 mm, ＜10 mm	5	7.6
≥10 mm, ＜15 m	36	55.4
TST average diameter ≥15 mm	22	33.8
IGRA-positive	35	53.8
Pathogen-positive		
CSF smear	4	6.1
CSF Xpert MTB/RIF	8	12.3
CSF TB culture	27	41.5
Cranial MRI		
Meningeal enhancement	42	64.62
Nodules, granulomas	30	46.15
Hydrocephalus	11	16.92
Skull base adhesions	38	58.46
Cerebral infarct foci	9	13.85
Cerebral edema	2	3.08

The head MRI results of 65 TBM patients showed 42 cases of meningeal enhancement, 30 cases of nodule and granuloma formation, 38 cases of skull base adhesion, 11 cases of meningeal thickening, 11 cases of hydrocephalus, 9 cases of cerebral infarction, and 2 cases of cerebral edema (Table 2).

Among the 65 CM patients included in the study, 51 underwent head CMI, which showed meningeal enhancement in 27 cases, nodule and granuloma formations in 27 cases, hydrocephalus in 3 cases, cerebral infarction in 4 cases, and cerebral edema in 5 cases (Table 3).

**TABLE 3:** Cranial MRI in CM patients

Cranial MRI	*n*	%
Meningeal enhancement	27	52.94
Nodules, granulomas	27	52.94
Hydrocephalus	3	5.88
Cerebral infarct foci	4	7.84
Cerebral edema	5	9.80

### Comparison of Igs in the TBM group before and after treatment

The levels of CSF IgG [331.51 (164.85, 645.00) vs 56.50 (31.98, 92.91) ng/mL, *Z* = −5.981, *P* < 0.001], IgM [22.38 (8.52, 40.18) vs 1.91 (1.06, 4.03) ng/mL, *Z* = −5.981, *P* < 0.001], and IgA [64.11 (21.44, 115.48) vs 4.61 (2.87, 13.98) ng/mL, *Z* = −6.144, *P* < 0.001] were decreased compared with those before treatment (Table 4).

**TABLE 4:** Comparison of Igs in the TBM group before and after treatment^*a*^

Items	Before treatment	After 24 weeks of treatment	*Z*	*P*
CSF IgG (ng/mL, *x* ± *s*)	331.51 (164.85, 645.00)	56.50 (31.98, 92.91)	−5.981	<0.001
CSF IgM (ng/mL, *x* ± *s*)	22.38 (8.52, 40.18)	1.91 (1.06, 4.03)	−5.981	<0.001
CSF IgA (ng/mL, *x* ± *s*)	64.11 (21.44, 115.48)	4.61 (2.87, 13.98)	−6.144	<0.001

^*a*^ TBM, tuberculous meningitis; CSF, cerebrospinal fluid.

### The diagnostic value of CSF albumin and Igs for TBM

The results showed that CSFs IgG, IgM, and IgA had predictive value for TBM. The areas under the curve (AUCs) of CSFs IgG, IgM, and IgA in predicting TBM were 0.736 (95% CI: 0.719–0.824), 0.729 (95% CI: 0.702–0.814), and 0.711 (95% CI: 0.695–0.838), respectively. The combined predictive value of the three indicators was the highest, with an AUC of 0.831 (95% CI: 0.774–0.881) (Table 5).

**TABLE 5:** Predictive values of Igs for TBM^*a*^

Items	AUC	95% CI	Sensitivity (%)	Specificity (%)
CSF IgG	0.736	0.719–0.824	70.61	71.70
CSF IgM	0.729	0.702–0.814	71.44	73.48
CSF IgA	0.711	0.695–0.838	72.01	73.82
Combined prediction	0.831	0.774–0.881	85.24	83.73

^*a*^ TBM, tuberculous meningitis; CSF, cerebrospinal fluid.

### Logistic regression analysis of CSF Igs and prognosis of TBM

The prognosis of patients was used as the dependent variable (good = 1; poor = 0), and factors with statistically significant differences in univariate analysis were used as independent variables (the original values of CSF Igs were included) to construct a logistic regression analysis model. The results of regression analysis showed that high CSF IgG (OR = 4.796, 95% CI: 2.575–8.864), high CSF IgM (OR = 3.456, 95% CI: 2.757–5.754), and high CSF IgA (OR = 4.371, 95% CI: 2.731–5.856) were risk factors for poor prognosis in TBM patients (Table 6).

**TABLE 6:** Logistic regression analysis of CSF Igs and prognosis of TBM^*a*^

Influence factors	S.E	Wald *χ^2^*	*P* value	OR	OR (95% CI）
CSF IgG	1.345	6.234	0.001	4.796	2.575–8.864
CSF IgM	1.256	6.785	0.001	3.456	2.757–5.754
CSF IgA	1.978	6.743	0.001	4.371	2.731–5.856

^*a*^ TBM, tuberculous meningitis; CSF, cerebrospinal fluid.

### Multiple linear regression analysis of CSF Igs and cranial MRI manifestations in the TBM group

Cranial MRI findings in TBM patients were used as dependent variables (1 = hydrocephalus, 2 = cerebral infarction, and 3 = granuloma, nodules, and enhancement), and variables with statistically significant differences in univariate analysis were used as independent variables to conduct a stepwise regression analysis, with CSF Ig levels included as continuous variables. These independent variables were added or removed using a stepwise regression method (*α*_entry_=0.05, *α*_removal_=0.1), and influencing factors with interaction were eliminated. It was found that no variables were eliminated during the entry and removal process and a regression model was constructed with *R*^2^ = 0.542, indicating that CSFs IgG, IgM, and IgA levels explained 54.2% of the cranial MRI manifestations in TBM patients (*F* = 65.392, *P* < 0.001). These results demonstrated that the dependent variables, i.e., MRI manifestations of the TBM patients, were well fitted with the three independent variables; the Durbin–Watson index was 1.995, suggesting that there was no correlation between these independent variables in the model; all *P* values of the significance test of the three independent variables in the model were <0.05, indicating the that the three independent variables were statistically significant in the model and should be retained; and the visual information fidelity (VIF) values of the three independent variables were <10, suggesting no collinearity among the variables. The multiple linear regression equation obtained by fitting was as follows:


$$Y=5.833+0.433X_{1}+0.322X_{2}+0.418X_{3}\;.$$

According to the partial regression coefficients in the model, the influence degree of three independent variables on the MRI manifestations of TBM patients was determined as CSF IgA > CSF IgG > CSF IgM (Table 7).

**TABLE 7:** Regression model of cranial MRI manifestations in TBM patients^*a*^

Variables	Standard error	Partial regression coefficient	*P*	95% CI	VIF
Constants	5.833	–	＜0.001	–	–
CSF IgG	1.323	0.433	＜0.001	0.365–0.722	2.002
CSF IgM	1.319	0.332	＜0.001	0.165–0.422	2.057
CSF IgA	1.520	0.418	＜0.001	0.355–0.512	3.064

^*a*^ *R* = 0.736, *R*^2^ = 0.542, adjusted *R*^2^ = 0.510, *F* = 65.392, *P* < 0.05. MRI, magnetic resonance imaging; TBM, tuberculous meningitis; CSF, cerebrospinal fluid, –; Not able to perform statistical analysis.

## DISCUSSION

In clinical practice, CNS infection is diagnosed based on medical history, imaging examination, and CSF tests before the pathogenic results are available. If there are no specific clinical manifestations, additional analysis is needed to improve the diagnostic accuracy (22). In the diagnosis of viral meningitis, it is generally believed in clinical practice that long T1 and T2 signals on MRI are the most diagnostic signs (23), which are similar to pathological changes of cerebral edema involving one or both insula, orbital surface of the frontal lobe, and the temporal lobe, with normal basal ganglia. In suppurative meningitis, basal meningeal enhancement is rarely seen on enhanced scan due to the rapid onset of this disease, and only a small number of patients develop communicating hydrocephalus, with organization and adhesion of exudate as the inducing factor (24). In the diagnosis of TBM, it is believed that basilar lacunar lesions, basal cistern/lateral fissure enhancement, and hydrocephalus on MRI are three suggestive imaging signs (25). CM usually has symptoms of meningitis, and no obvious hydrocephalus and basal meningeal enhancement are seen on imaging examination, or only enhanced cryptococcal tumor or non-enhanced gelatinous cysts are shown. In some special cases, the perivascular space is occasionally enlarged (26). In the present study, the findings on MRI in the TBM group mainly included hydrocephalus, granuloma, nodules, and enhancement, and the findings in the CM group mainly included granuloma, nodules, and enhancement, which were consistent with the findings of the studies described above.

CSF test is commonly used in the differential diagnosis of CNS infection to obtain pathogenic evidence and can serve as the gold standard for the diagnosis of these diseases (27). However, pathogenic examination is time-consuming and involves various influencing factors, making it difficult to obtain accurate evidence in the early clinical stage. Therefore, it is necessary to analyze and use common indicators (28). In the present study, comparison of CSFs IgG, IgM, and IgA between the TBM group and the CM group revealed that CSFs IgG, IgM, and IgA were increased in the TBM group compared with those in the CM group, and the differences were statistically significant (*P* < 0.05), suggesting that the BBB was more damaged in TBM than in CM. Before treatment, the CSFs IgG, IgM, and IgA of patients in the TBM group were higher than those in the healthy population (29) and lower than the corresponding values of patients with multiple sclerosis (30) and neurosyphilis (31). Comparison of CSFs IgG, IgM, and IgA in the TBM group also showed significant differences before and after treatment (*P* < 0.05). With the absorption of the lesions and the recovery of the disease, these indicators gradually decreased and returned to normal. The correlation analysis between CSFs IgG, IgM, and IgA and cranial MRI manifestations (hydrocephalus, cerebral infarction, granuloma, nodules, and enhancement) in the TBM group showed that higher levels of CSFs IgG, IgM, and IgA indicated more chance of granulomas, nodules, and enhancement and less chance of hydrocephalus on cranial MRI. It can be understood that if the CSFs IgG and IgM are higher, the intracranial TB focus is mainly hyperplasia, and on the contrary, it is mainly exudation. Therefore, based on the level of Ig in the CSF, doctors can determine the nature of the lesion (proliferative or exudative) and adjust the treatment plan accordingly. For example, in addition to anti-TB treatment, appropriate immunomodulatory treatment can be given to patients with high Ig levels.

The level of CSF Igs (also known as special globulins) is very low and increases to certain degrees when inflammation and tissue and BBB damages occur in CNS (32). The increase of CSF proteins often indicates the increased brain barrier permeability (33). In addition, when CNS is severely infected, the activation and synthesis of immune cells also increase. CSF Igs, mainly IgA, IgG, and IgM, are derived from serum under normal conditions. IgA is the most important antibody in CSF and significantly increases when local infection occurs. With a small molecular weight, IgG can relatively freely pass through the BBB, and the level of IgG in CSF is relatively high. IgM has a large molecular weight and is difficult to penetrate the BBB, with a low concentration in CSF. However, the permeability of the BBB will increase with the contribution of inflammation. Studies have shown that the increase of CSFs IgG and IgA after CNS infection is more significant in TBM patients than that in other types of CNS infectious diseases (33), and the levels of CSFs IgG and IgM can reflect the degree of BBB damage and help in the evaluation of the outcomes of the disease (34). These are consistent with the findings of the present study.

In studies on the correlation between CSF Igs and the pathogenesis of CNS diseases available (35), more researchers are focused on autoimmune encephalitis (33), and reports on CNS infectious diseases are relatively rare. Many studies (36) found that CSF Igs can be used as an indicator for differential diagnosis of CNS infectious diseases when there is no pathogenic evidence for definite diagnosis.

TBM is very similar to CM in terms of pathogenesis, clinical manifestations (such as meningeal irritation sign, consciousness disorder, and cranial nerve damage), routine CSF biochemistry (decreased glucose and increased CSF proteins), and cranial MRI manifestations (both diseases are manifested as granuloma, meningeal enhancement, hydrocephalus, and cerebrovascular lesions) (34). However, comparison of CSF Igs before treatment between TBM and CM shows that the concentration of CSF Igs was significantly higher in TBM patients than that in CM patients, indicating that the BBB was more severely damaged in TBM than in CM. On this basis, CSF Igs can be used as one of the diagnostic indicators to distinguish TBM from CM in the absence of CSF pathogenic evidence.

There were certainly limitations in the present study. Firstly, this was a retrospective study with limitations in the inclusion criteria, clinical data selection, and processing. Secondly, only patients admitted to our hospital were selected as study subjects, which may lead to biased results. Future in-depth studies with large sample sizes and complete data are necessary to provide a more accurate and practical basis for effective treatment of this disease.

### Conclusion

In summary, CSFs IgG, IgM, and IgA can be used as a routine monitoring indicator for TBM and CM patients and offer valuable references for the differential diagnosis and efficacy evaluation of these diseases. Higher levels of CSFs IgG, IgM, and IgA suggest more possibility of TBM and worse prognosis and MRI manifestations.

## Data Availability

The data sets used and analyzed during the current study are available from the corresponding author on reasonable request.
